# Lignocellulose degradation mechanisms across the Tree of Life

**DOI:** 10.1016/j.cbpa.2015.10.018

**Published:** 2015-11-14

**Authors:** Simon M Cragg, Gregg T Beckham, Neil C Bruce, Timothy DH Bugg, Daniel L Distel, Paul Dupree, Amaia Green Etxabe, Barry S Goodell, Jody Jellison, John E McGeehan, Simon J McQueen-Mason, Kirk Schnorr, Paul H Walton, Joy EM Watts, Martin Zimmer

**Affiliations:** 1 School of Biological Sciences, University of Portsmouth, King Henry Building, King Henry 1st St., Portsmouth PO1 2DY, UK; 2 National Renewable Energy Laboratory, National Bioenergy Centre, Golden, CO 80401 USA; 3 University of York, Department of Biological Sciences, Centre for Novel Agricultural Products, York YO10 5DD, UK; 4 Department of Chemistry, University of Warwick, Coventry CV4 7AL, UK; 5 Ocean Genome Legacy, Marine Science Center, Northeastern University, Boston, MA, USA; 6 Department of Biochemistry, University of Cambridge, Hopkins Building, Tennis Court Road, Cambridge CB2 1QW, UK; 7 Department of Sustainable Biomaterials, 216 ICTAS II Bldg., Virginia Polytechnic Institute and State University (Virginia Tech), Blacksburg, VA 24061, USA; 8 Department of Plant Pathology, Physiology and Weed Science, Virginia Polytechnic Institute and State University (Virginia Tech), Blacksburg, VA 24061, USA; 9 Novozymes AS, DK-2880 Bagsvaerd, Denmark; 10 Department of Chemistry, University of York, York YO10 5DD, UK; 11 Leibniz-Center for Tropical Marine Ecology (ZMT) GmbH, Fahrenheitstrasse 6, 28359 Bremen, Germany

## Abstract

Organisms use diverse mechanisms involving multiple complementary enzymes, particularly glycoside hydrolases (GHs), to deconstruct lignocellulose. Lytic polysaccharide monooxygenases (LPMOs) produced by bacteria and fungi facilitate deconstruction as does the Fenton chemistry of brown-rot fungi. Lignin depolymerisation is achieved by white-rot fungi and certain bacteria, using peroxidases and laccases. Meta-omics is now revealing the complexity of prokaryotic degradative activity in lignocellulose-rich environments. Protists from termite guts and some oomycetes produce multiple lignocellulolytic enzymes. Lignocellulose-consuming animals secrete some GHs, but most harbour a diverse enzyme-secreting gut microflora in a mutualism that is particularly complex in termites. Shipworms however, house GH-secreting and LPMO-secreting bacteria separate from the site of digestion and the isopod *Limnoria* relies on endogenous enzymes alone. The omics revolution is identifying many novel enzymes and paradigms for biomass deconstruction, but more emphasis on function is required, particularly for enzyme cocktails, in which LPMOs may play an important role.

## Introduction

Land plants direct most photosynthetically fixed carbon into lignocellulose, a composite of the polymers cellulose, hemicellulose, pectin and lignin. During the life of the plant, this complex matrix provides structural integrity, and resistance to herbivores and pathogens, so most lignocellulosic biomass is processed by saprophytes and detritivores in detrital food webs. Biomass can be used as a feedstock for biofuel generation, but is recalcitrant to enzymatic processing due to barriers to enzyme access that arise from the paracrystallinity of cellulose, the complexity of the hemicellulose coating of cellulose microfibrils, and the interpenetration and encapsulation of polysaccharide components by lignin. In industrial processes, recalcitrance is overcome by severe chemical and physical pre-treatments, but organisms achieve lignocellulose deconstruction under physiologically tolerable conditions. To assist the prospecting of biodiversity for lignocellulolytic mechanisms with potential for biotechnology applications, a discussion meeting was held in September 2013 at the Linnean Society in London, which reviewed the vast array of mechanisms across the Tree of Life. This article captures and updates the diverse chemical and organismal perspectives brought to the subject by the participants in the meeting.

## Diversity of deconstruction mechanisms

Organisms achieve lignocellulose deconstruction in diverse ways. Oxidative attack, hemicellulases and, in animals, mechanical disruption all reduce recalcitrance, which improves access for depolymerising enzymes. Information on carbohydrate-active enzymes and substrate-binding proteins (carbohydrate-binding modules) is collated within the CAZy database [1]. Peptide pattern recognition (PPR) has recently been used to assist the classification of GH and AA families into subfamilies, based on predicted function, and to provide a tool for mining genome data for new enzymes [2]. Here we focus on the CAZy categories of glycoside hydrolases (GHs) and Auxiliary Activities (AAs) — redox enzymes that act with GHs, often in a synergistic manner.

## Enzymatic depolymerisation of cellulose and hemicelluloses

**Figure 1:**
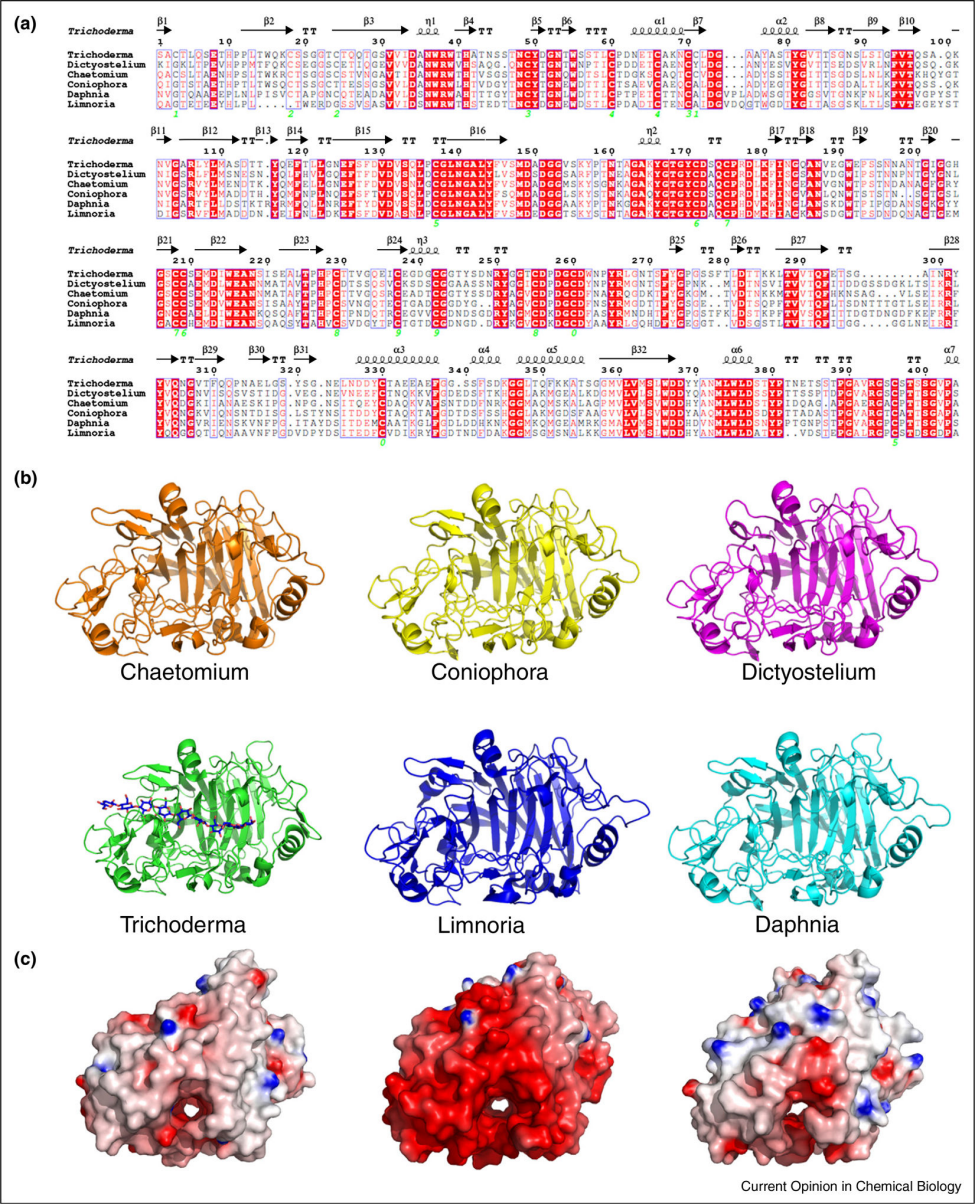
Variability within the CAZy Family GH7. Conservation of GH7 family enzymes from across the Tree of Life. **(a)** Primary sequences of the core regions of *Trichoderma reesei, Dictyostelium discoideum, Chaetomium thermophilum, Coniophora puteana, Daphnia pulex* and *Limnoria quadripunctata* were aligned with CLUSTALW [69]. Sequences were rendered using ESPRIPT [70]. Conserved regions are marked in blue boxes, identity by white text on red background and similarity with red letters. Secondary structure elements are based on the *T. reesei* structure (PDB ID: 1CEL) with helices displayed as coils, β-strands as arrows, strict β-turns as TT letters and strict α-turns as TTT letters. Green numbers for cysteine residues indicate their pairing in disulphide bridges as known from the structures of the *Trichoderma* and *Limnoria* enzymes. **(b)** Homology models generated with SWISSMODEL [71] rendered as cartoons with PyMOL (Schrödinger, LLC) were compared to the X-ray structures of *T. reesei* (PDB ID: 1CEL) and *L. quadripunctata* (PDB ID: 4IPM) revealing a high conservation prediction of the structural fold. Differences in loop regions correspond with regions of low identity in part (a). Note the size of the protein relative to a cellulose chain bound in the tunnel of *T. reesei* GH7 (Glc9 oligosaccharide from PDB ID: 4C4C [72]). **(c)** Electrostatic surface mapping of *T. reesei, L. quadripunctata* and *D. pulex* demonstrates that while the backbone is highly conserved, there is a striking evolution of surface properties corresponding to environment. Electrostatic potential between −*7kT/e* and *7kT/e* was plotted with DELPHI [73] as a coloured gradient from red (acidic) to blue (basic). The fresh water *Daphnia* has a relatively neutral surface coat similar to that of the *Trichoderma* fungus. By contrast, the other crustacean, *L quadripunctata*, has a highly acidic surface coat, presumably adapted for digestive processes within the marine environment.

The enzymatic degradation of cellulose and hemicellu-lose is accomplished in Nature via the collective action of multiple carbohydrate-active enzymes, typically acting together as a cocktail with complementary, synergistic activities and modes of action [3••]. GHs are the primary enzymes that cleave glycosidic linkages present in cellulose and hemicellulose. GHs are assisted in their function by polysaccharide esterases that remove methyl, acetyl and phenolic esters, allowing the GHs to function on hemicelluloses [4]. In some cases, polysaccharides are also depolymerised by the action of polysaccharide lyases [4]. Across the Tree of Life, the GH cocktail composition is greatly dependent on the kingdom of the cellulolytic organism, the evolutionary pressure the organism has faced, and the environmental niche wherein it resides. For example, filamentous cellulolytic fungi produce GH Family 7 enzymes, which are potent cellobiohydrolases [3••], but in prokaryotes this function is provided by other families such as GH48. Until recently, it was also long thought that GH7 enzymes were only found in fungi, but recent studies have revealed their existence in other eukaryotic kingdoms of life [5,6]. Despite phylogenetic diversity, remarkable sequence and structural similarities occur within this GH family (e.g., Figure 1a,b), though the enzyme surface properties may be markedly different (Figure 1c). Greater diversities of sequence and function are found within other GH families.

**Figure 2:**
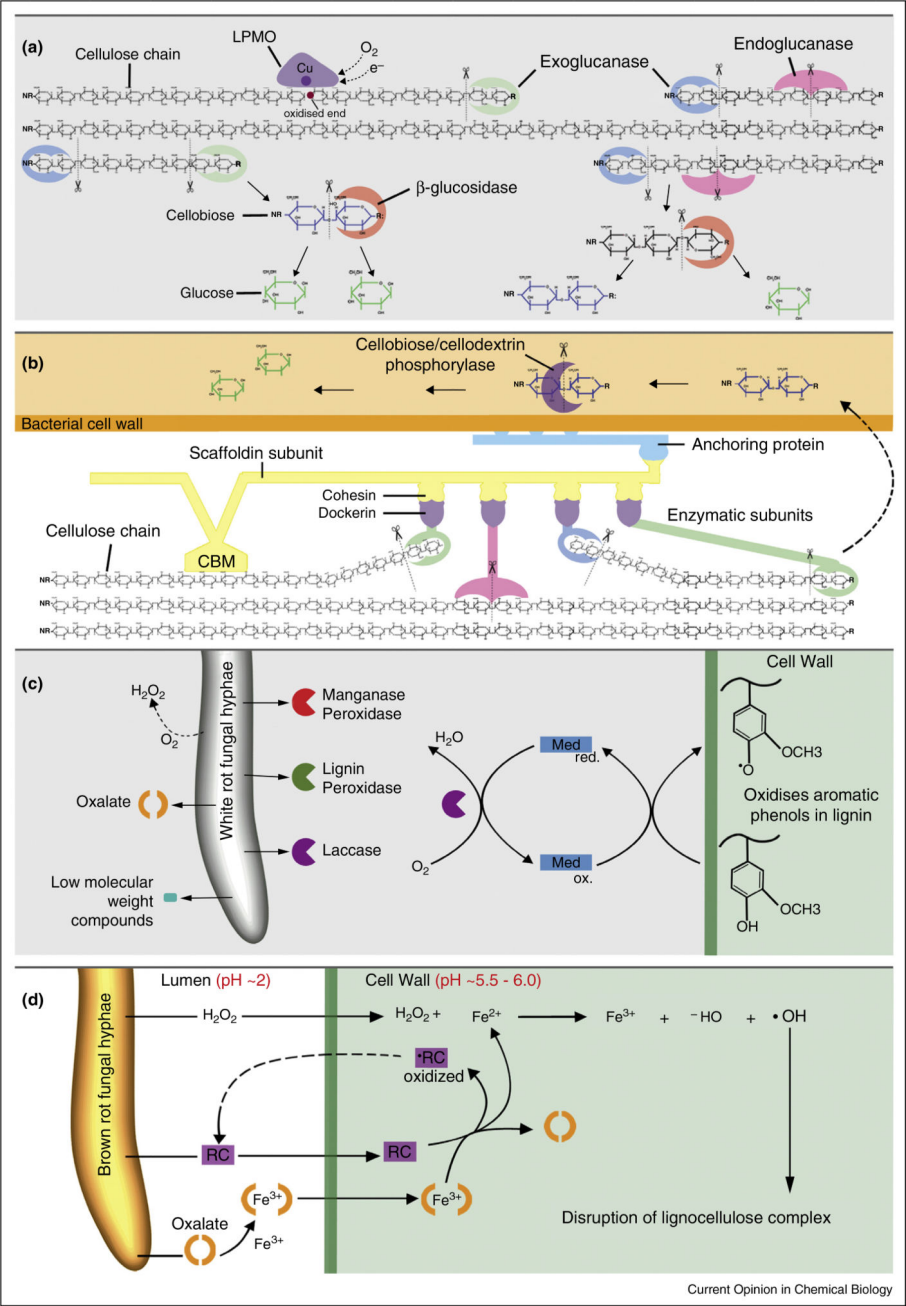
Schematics of microbial mechanisms of lignocellulose degradation. **(a)** Aerobic cell-free cellulase system employed by many bacteria and fungi. Cellulose is hydrolysed via the synergistic interaction of individual GH and LPMO (AA9 or 10) secreted enzymes (enzyme reaction sites only shown on the cartoon, not to scale). NR-, non-reducing ends; -R, reducing ends. **(b)** Anaerobic ‘cellulosome’ mechanism. The cellulosome is a complex attached to the bacterial cell wall via an anchoring subunit. The complex consists of enzymes capable of cellulose hydrolysis attached to a scaffoldin subunit which anchors the bacterial cell and enzymes to the substrate via a carbohydrate binding module (CBM). **(c)** Lignin degradation by white rot fungi which secrete extracellular enzymes such as peroxidases and laccases and their low molecular weight co-factors to generate oxidative radical species which catalyse the oxidation of lignocellulose. Lignin peroxidases oxidise non-phenolic aromatic moieties while manganese peroxidases and laccases oxidise phenolic subunits. Laccase can act upon non-phenolic subunits of lignin by the inclusion of a mediator (Med). **(d)** Disruption of the lignocellulose complex by brown rot fungi using the chelator-mediated Fenton system (CMF). Fungal hyphae in the lumen of plant cells produce iron-reducing compounds (RC), hydrogen peroxide (H_2_O_2_) and oxalic acid. The oxalic acid binds Fe^3+^ as a complex which diffuses into cell wall along with H_2_O_2_ and RC. With the pH change, RC sequesters Fe^3+^ from the Fe-oxalate complex and reduces it to Fe^2+^. Fe^2+^ then reacts with H_2_O_2_ (Fenton reaction) and produces hydroxyl radicals (•OH) which disrupt the lignocellulose. Modified from (a) Refs. [74,11••] and (d) Ref. [14].

Cellulolytic enzymes can also be arranged in multiple domain architectures. For example, some rumen bacteria and fungi employ a large, multi-modular cellulosome approach with many catalytic units on a large scaffold [7], whereas many prokaryotic and eukaryotic species employ free enzyme paradigms with single catalytic units able to diffuse and act independently (Figure 2a,b). Some enzymes have interpolation between these paradigms wherein a single protein contains more than one active site [8•], for example, a multimodular enzyme with GH5, GH6, CBM5 and CBM10 domains has been found [9•].

## Oxidative polysaccharide depolymerisation

Recently, a new oxidative enzymatic paradigm was discovered for cleavage of polysaccharide linkages [10]; these enzymes have been termed lytic polysaccharide monooxygenases (LPMOs), but some were originally classified as GH Family 61 cellulases and others, Family 33 Carbohydrate-Binding Modules. Cellulose-degrading LPMOs are now assigned to AA family 9, which contains fungal enzymes, and AA10 with predominantly bacterial enzymes [11••]. LPMOs can act on crystalline cellulose [11••], but also hemicelluloses [12]. They act by direct oxidative attack on the polymer chains (Figure 2a) through a flat active site with a centrally located copper atom [13•]. Non-enzymatic deconstruction of the cellulose can also be demonstrated, including iron-dependent Fenton chemistries found in the brown rot wood-degrading fungi [14].

## Lignin depolymerisation

Lignin is a heterogeneous, alkyl-aromatic polymer found in plant cell walls formed from three aromatic alcohols that differ in their extent of methoxylation. Multiple strategies exist in Nature for the modification of lignin, though a much more limited range of organisms can achieve lignin degradation than cellulose degradation. White rot basidiomycetes and some ligninolytic bacteria serve as the primary degraders of lignins via the action of secreted oxidative enzymes such as peroxidases and laccases [15,16••] (Figure 2c), producing a pool of heterogeneous aromatics. These are ultimately metabolized by the secreting organism or other microbes. Brown rot fungi, which have no lignin degrading enzymes, employ small molecule reactive species to depolymerize lignin (Figure 2d), cleave the propyl side chain, and also demethoxylate the ring before repolymerizing the material elsewhere as a means of freeing the cellulosic components and generating greater access for deconstruction [14]. The modified lignin is not metabolized by brown rot fungi and instead persists in the soil.

## Diversity of lignocellulose-degrading organisms

**Figure 3:**
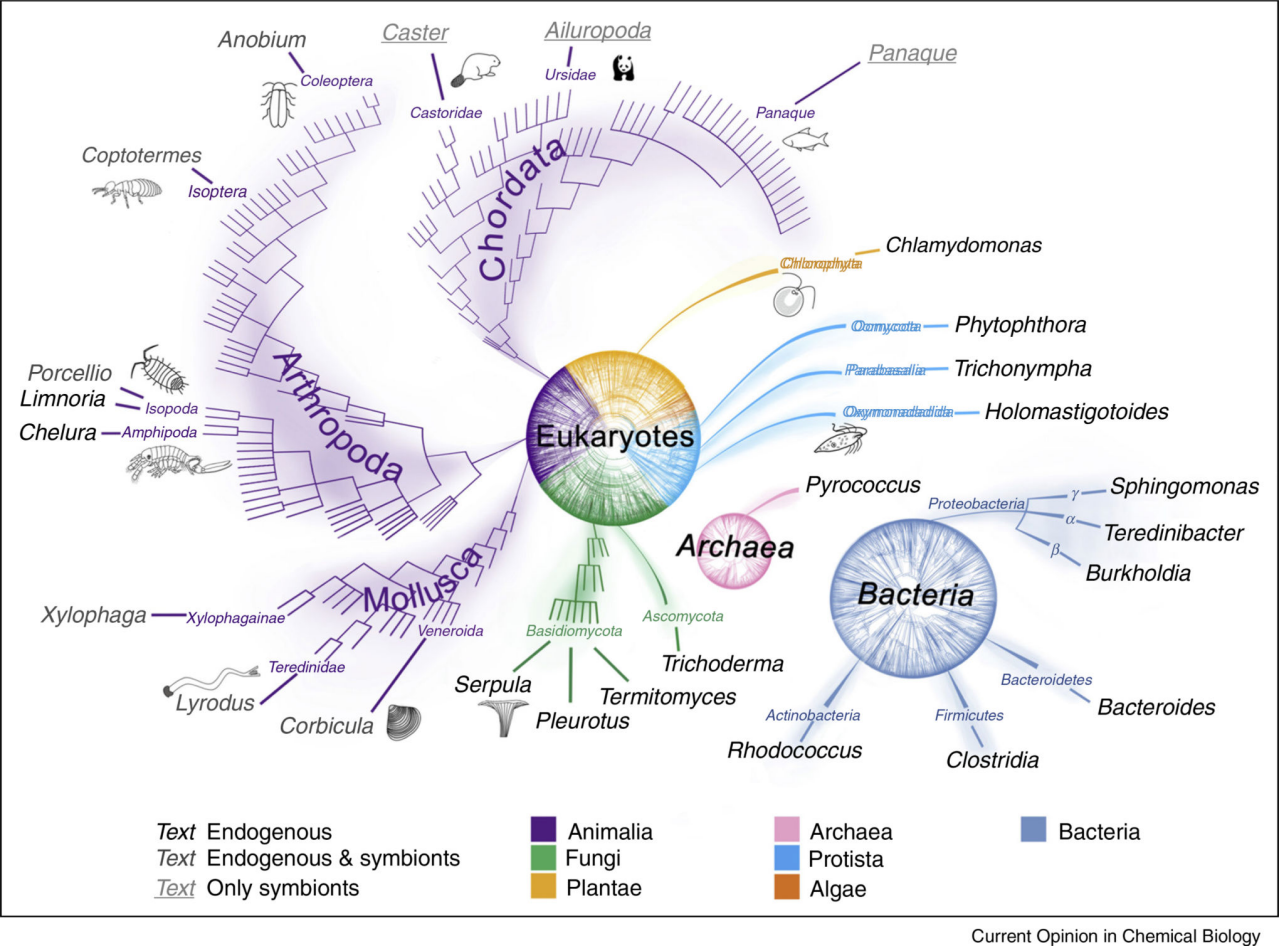
The sparse and localised distribution of selected organisms capable of lignocellulose or cellulose degradation mapped onto the Tree of Life, with highest taxonomic ranks colour-coded as shown in key. Genus names of organisms degrading lignocellulose using endogenous enzymes shown in bold, those with endogenous plus symbiont-derived enzymes shown printed pale and those with only symbiont-derived enzymes shown underlined.

Cellulose is generated by a diversity of marine organisms so cellulose breakdown is probably to have an ancient origin. The evolution of lignin degradation, however, coincided with the decline in organic carbon burial at the end of the Permian [17]. Land plants appeared after most the major branches of the Tree of Life had already diverged, so the ability to deconstruct lignocellulose has multiple origins and has continued to evolve in diverse smaller branches widely, but sparsely dispersed across the Tree of Life (Figure 3). For example, the ecologically important insect-protist symbiosis, which facilitates lignocellulose digestion, emerged in the late Jurassic [18••] and wood digestion aided by bacterial mutualists was a feature of the last common ancestor of the bivalve families Teredinidae and Xylophagainae [19]. In Nature, symbioses and consortia of organisms with complementary enzymes feature widely in breakdown of bulk biomass. Deconstruction is achieved under a wide range of (sometimes extreme) environmental conditions, particularly of pH, redox potential, temperature, and pressure. This range is reflected in the diversity of the organisms involved.

## Prokaryotes

Recent developments in powerful meta-omic techniques are making it possible to mine the incredible genetic diversity of prokaryotic communities of lignocellulose-enriched environments, such as compost, for new robust lignocellulose degrading enzymes that could potentially perform well under industrial conditions. Comparative meta-transcriptomic analysis has recently been used to identify highly expressed genes in compost-derived microbial communities capable of degrading rice straw under high loading conditions [20]. Studies on lignocellulose degrading microorganisms in complex communities, using meta-genomics and meta-proteomics, are revealing the structure and roles of individual community members and how they respond to changes in environmental conditions such as nutrient availability at functional and genetic levels.

**Figure 4:**
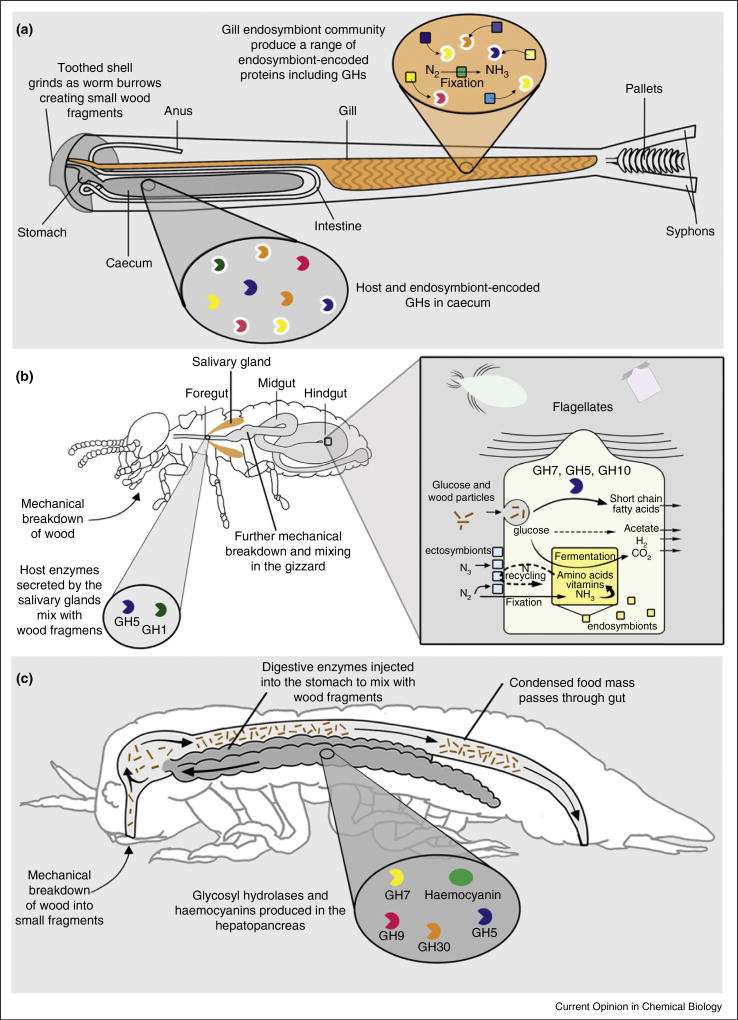
Examples of mechanisms employed by animals in lignocellulose degradation. **(a)** Shipworms bore into the wood using a shell with toothed ridges, creating small wood fragments which are ingested. Shipworms house dense communities of endosymbiotic bacteria in an internal region of the gill referred to as the gland of Deshayes. Some of the endosymbiont lignocellulose degrading enzymes are selectively translocated from gill to gut where they mix with host enzymes to digest the wood fragments. **(b)** In the termite foregut, wood particles are mixed with enzymes excreted by salivary glands and further comminuted in the gizzard. Glucose released in the midgut is resorbed, whereas the partially digested wood particles pass through to the hindgut. They are phagocytized by cellulolytic flagellates, which hydrolyse the remaining polysaccharides using cellulases and hemicellulases that are secreted into their digestive vacuoles. The microbial fermentation products (which are mainly short-chain fatty acids) are resorbed by the host, and the lignin-rich residues are voided as faeces. **(c)** Limnoriids have a simple straight digestive tract with two paired posteriorly directed hepatopancreas lobes (caeca) which join the stomach in the head region. As the crustacean eats, it mechanically breaks down the wood into small fragments. In the stomach the small wood fragments mix with the digestive enzymes secreted by the hepatopancreas. The wood fragments are compressed together and the indigestible components are excreted as faecal pellets.Modified from (a) Ref. [50••], (b) Ref. [18••] and (c) Ref. [6].

Meta-omics also yields new insights into the complex inter-relationships in gut-resident microbial consortia. Termites provide particularly intriguing examples of digestive mutualism [21]. In lower termites, bacteria and archaea live in the cytoplasm and on the external surfaces of gut-resident, wood-particle-phagocytosing flagellates, but also in the viscous gut fluids. Bacteriodetes, Firmicutes, Spirochaetes, Proteobacteria and Elusibac-teria are prominent members of this microbiota which participate in the pathways leading to conversion of biomass to methane, hydrogen and acetate [18••] (Figure 4b). Over 4700 bacterial phylotypes have been detected by 16S rRNA probes in the lower termite *Reticulitermes* [22]. The hindgut of higher termites contains only prokaryotes and these promote the breakdown of wood particles pre-treated by enzymes from the termite. Hindgut fluids have low cellulolytic activity, but strong cellulolytic activity is found in wood particles and the bacteria associated with them [23].

A number of soil bacteria have been identified that are able to oxidise lignin, the majority of which fall into the Actinobacteria, α-Proteobacteria or γ-Proteobacteria, members of which have also been found in termite guts and wood-boring insects [15]. The enzymes responsible for degradation of lignin in prokaryotes were until recently poorly understood, but peroxidases from the dye-decolorising peroxidase family have been shown to be active for oxidation of Mn(II) and β-aryl ether lignin model compounds in Gram-positive actinobacteria *Rhodococcus jostii* RHA1 [24] and *Amycolatopsis* sp. 75iv2 [25], and in Gram-negative γ-proteobacterium *Pseudomonas fluorescens* Pf-5 [26]. Bacterial laccases have also been shown via gene deletion to be required for production of acid-precipitable lignin in *Streptomyces* A3(2) [27]. Glutathi-one-dependent β-etherase enzymes that catalyse stereospecific cleavage reactions on β-aryl ether lignin model compounds have also been characterised from *Sphingobium* SYK-6 [28•], though the role of these enzymes and their contribution to lignocellulose degradation remains to be characterised. Laccase and peroxidase activity has been identified and characterised in a range of bacteria grown on biomass-derived lignin [29].

Archaea are also found in composts [30] and termite guts [22,31], but their mechanisms of lignocellulose deconstruc-tion are less well explored. Some Archaea can degrade lignocellulose at high temperature [32,33]. An endogluca-nase GH12 has been identified in the archaeon *Pyrococcus* [33]. Five genes encoding laccase enzymes that might oxidise lignin have been identified in Archaea, three in the Halobacteriales, and one in the Thermoproteales [31].

Free-living, wood degrading prokaryotes from marine sources are categorized into tunnelling or erosion bacteria, distinguished by their distinct patterns of plant cell-wall degradation [34]. Tunnelling bacteria are gram negative rods and erosion bacteria are assigned to the *Cytophaga-Flavobacteria* group: neither type have been grown in pure culture so their evidently independent action is poorly understood, but the rate is slow compared with fungal decay [34], leaving lignin little altered while degrading cellulose and hemicellulose [35]. Wood exposed in deep water recruits characteristic assemblages of pressure-tolerant bacteria which are distinct from those found in faecal material produced by borers feeding on the wood [36].

## Single celled eukaryotes and protists

Endogenous cellulases have been detected in some free-living protists. The genome of the slime mould *Dictyostelium* encodes a putative GH7 cellobiohydrolase [37] and the chlorophyte *Chlamydomonas* is capable of breaking down extracellular cellulose using an endoglucanase [38]. The dinoflagellate *Alexandrium* generates a cellulase similar to one from termite symbionts, but this probably assists cell division rather than digestion [39]. However, the pathogenic oomycete, *Phytophthora* generates a suite of cell wall degrading enzymes that target hemicellulose and cellulose, including members of GH families 1, 5, 6, 7 and 10, and AA9 and 10 [40]. Lower termites host up to 19 species of flagellate parabasilian and oxymonadid protists in their paunch which phagocytose wood particles. These protists contain a plethora of enzymes in their digestive vacuoles: endoglucases, GH7-cellobiohydrolases, β-glucosidases, xylanases, mannanosidases and arabinosidases [18••].

## Fungi

Biomass degrading fungi rely on complex degradative machineries that generally catalyse two types of processes: first, direct enzymatic depolymerization, for example, by cellobiohydrolases and second, generation of oxidative species (e.g., radicals) that then act on the biomass. Categorization terminology is changing with new genomic information on the Basidiomycota suggesting that fungal species traditionally classed as white rot or brown rot may no longer fit neatly into these categories because of gradations both in the expression of metabolites and the resulting patterns of decay [41••]. Traditionally however, in typical white rot degradation, the fungi employ a mode of attack that is primarily enzymatic. Attack of the wood cell wall proceeds only from lignocellulose surfaces in white rot fungi because degradative enzymes are too large to penetrate the intact cell wall. The enzymes employed by the white rot fungi include a complete suite of cellulases, and these fungi also produce a suite of enzymes that can oxidise lignin components, including ligninase, manganese peroxidase, versatile peroxidase or laccase, or a combination of these (Figure 2c) [16••]. Some white rot fungi have also been shown to have large numbers of LPMO genes [42••].

Brown rot fungi have evolved multiple times from the predecessors of current white rot fungi and in these evolutionary advances, lignolytic enzyme systems and crucial types of cellulases have been lost [17]. A chelator-mediated Fenton (CMF) system (Figure 2d) has evolved to substitute for much of the cellulolytic enzyme machinery in at least three orders of brown rot fungi (Gloeophyllales, Polyporales and Boletales), thus generating an alternative efficient mechanism for depolymerization and biomodifi-cation of biomass [14,43]. The CMF system is unique among biological systems in being the only reported substrate deconstruction system based on oxygen radical chemistry that permits non-enzymatic deconstruction at a considerable distance (several microns) from the organism. The efficiency of the CMF system is thought to provide brown rot fungi advantages in exploiting ecological niches, and for example, these fungi have displaced white rot predecessors in the degradation of conifer wood.

Some ascomycete fungi can also degrade wood cell walls, forming chains of diamond-shaped cavities that generally follow the orientation of the S2 elementary fibrils, causing soft rot [34]. Soft rot fungi are known to produce a full complement of cellulolytic enzymes; however, their lignin degrading ability has been variably reported to contain unspecified extracellular peroxidases and oxidases that appear to be more limited in function than those isolated from white rot fungi.

## Animals

Many invertebrates express endogenous cellulases. Plant-parasitic nematodes, cockroaches and termites were among the first to be proven to carry cellulase genes, but more recently these genes (mostly of the families GH5, 9 and 45) have also been unambiguously demonstrated in other taxa, such as other insects [44], Gastropoda [45], Crustacea [6,46,47] and Annelida [48]. The lack of large digestive gut chambers (as known from ruminants and termites) for cultivation of microbial gut symbionts in many insects or crustaceans argues that endogenous cellulases are needed in these herbivorous and detritivorous animals. Overcoming recalcitrance is partially achieved by mechanical breakdown of substrate by mouth parts or shells.

Wood-boring teredinid bivalves (commonly called shipworms) ingest wood particles produced by the grinding action of their shells. They lack a conspicuous gut micro-biota [49] and instead, harbour endosymbiotic γ-proteobacteria within specialized cells in the gills. In the shipworm *Bankia setacea*, these bacteria produce lignocellulose-degrading enzymes that are selectively transported to the gut [50••]. These enzymes include representatives of GH families 5, 6, 9, 10, 11, 45 and 53 and carbohydrate esterase families 1, 3, 4, 6 and 15, as well as LPMOs from the AA10 family [9•]. This separation of bacterial residence from digestion may allow the capture of liberated sugars without competition from a resident gut microbiota (Figure 4a). The endosymbiotic bacteria have been shown to fix nitrogen in vivo and thus may help to complement the limited organic nitrogen sources in wood [51]. Deep sea relatives of shipworms — the Xylophagainae — have a similar symbiosis and breakdown mechanism, but one that is capable of operating at extreme pressures [19]. Endogenous GH 9, 10 and 45 enzymes have been detected in the digestive gland and crystalline style of the bivalve *Corbicula* which consumes particulate detritus from terrestrial plants [52]. The role of the crystalline style in breakdown of heavily lignified substrates remains to be elucidated and is a promising line of enquiry.

In termites, endogenous cellulases (produced in the salivary glands and midgut) are complemented by microbial enzymes produced by flagellates and bacteria in the hindgut [53] (Figure 4b), the latter also allowing partial access to cellulose fibres through oxidative breakdown of the embedding lignin matrix. The role of endogenous phenol oxidase-like enzymes in lignin degradation in other invertebrates remains unclear, but recent studies suggest an involvement of activated haemocyanin in phenol oxidation [6,54]. Hemicellulases have been demonstrated in crustaceans, of which at least laminarinases are endogenous [55]. In termites, hemicellulases (xylanase, galactanase) appear to be mostly of bacterial origin [53], though mannanase activity has been ascribed to a symbiotic protist of a termite [56].

Whilst most termites rely on gut-resident microbiota, sometimes resident within the cells or even nuclei of the flagellate protists [18••,^57^], members of the Macrotermitinae cultivate the basidiomycete fungus *Termitomyces* on termite faecal pellets formed into comb-like structures in their mounds. This fungus produces a wide range of GHs capable of hydrolysing complex polysaccharides. The termite workers host bacteria capable of digesting oligosaccharides released by the fungus [58].

The wood-consuming crustaceans *Chelura* (Amphipoda) and *Limnoria* (Isopoda) generate endogenous enzymes belonging to a number of CAZy families, with GH5, 7 and 9 members being most prominent in the transcriptome of the digestive gland [6,59•]. They, together with certain other crustaceans, are the only metazoans known to produce GH7 enzymes. They have digestive tracts devoid of resident microorganisms and thus lack the biologically structured gut chemistry found in termites. These organisms have an enzyme-reactor type of gut (Figure 4c) and offer an exciting model for examining enzyme function without the complication of microbial interactions.

Lignocellulose digestion is a rare dietary strategy in vertebrates, but a few terrestrial (e.g., beavers, pandas and porcupines) and aquatic vertebrates consume high levels of lignocellulose in their normal diet [60,61], but it is unclear whether this is obligate xylophagy, except in the case of pandas, which are surprisingly poorly adapted to their diet [62]. The microbiomes that facilitate lignocellulose digestion in vertebrates vary greatly and are now being investigated. Loricariid catfish are found predominantly in freshwater ecosystems of the Neotropics, and a subset — *Panaque* spp. are xylivorous. Using 16S rRNA gene analysis it was found that *P. nigrolineatus* GI tract possesses a microbial community comprising close relatives of microorganisms capable of cellulose degradation and nitrogen fixation [63]. Cellulose-degrading bacteria from this community have been characterised and found to exist in symbiosis with nitrogen-fixers within this vertebrate GI tract [64].

## Conclusions

The advent of omics technologies, coupled to heightened interest in biofuels motivated by the drive towards a sustainable energy future, has driven a rapid increase in our repertoire of lignocellulose-active genes and understanding of natural paradigms. Furthermore, recent discoveries in polysaccharide oxidation [11••], substrate binding paradigms [65•], enzyme domain architectures [8•,9•], synergies between enzymatic modes of action [66] and enzymes for lignin bond cleavage [28•] highlight the fact that many discoveries remain ahead of us. Our understanding of the deconstruction process at molecular and microscopic levels has been enhanced by innovative visualisation of degradation of experimental substrates [8•,^67^]. However, the development of detailed sequence–structure–function relationships for individual enzymes still lags behind, even for enzymes that are considered to be well characterised, such as fungal cellulases [3••] and hemicellulases [68•], and certainly in more recently discovered oxidative enzymes [11••] and those involved in lignin degradation [15]. Beyond understanding single enzymes, the ability to understand how cocktails of enzymes work together synergistically will be undoubtedly crucial to understanding how to harness paradigms observed in Nature and to optimize these to industrial conditions. The ability of organisms and microbial communities to adjust their enzyme cocktails to different substrates almost certainly contains some clues. Tolerance to specific conditions may guide selection of enzymes for biotechnological exploitation [59•]. A more complete understanding and exploitation of the evolutionary inventions offered by the Tree of Life to overcome recalcitrance will ultimately be achieved by combining tools from diverse fields including microbiology, zoology, biochemistry, omics approaches, synthetic biology, advanced imaging and substrate characterisation [3••].

## References

[1] Lombard V, Ramulu HG, Drula E, Coutinho PM, Henrissat B (2014). The carbohydrate-active enzymes database (CAZy) in 2013. Nucleic Acids Res.

[2] Busk PK, Lange M, Pilgaard B, Lange L (2014). Several genes encoding enzymes with the same activity are necessary for aerobic fungal degradation of cellulose in Nature. PLoS One.

[3••] Payne CM, Knott BC, Mayes HB, Hansson H, Himmel ME, Sandgren M, Ståhlberg J, Beckham GT (2015). Fungal cellulases. Chem Rev.

[4] van den Brink J, de Vries RP (2011). Fungal enzyme sets for plant polysaccharide degradation. Appl Microbiol Biotechnol.

[5] Eichinger L, Pachebat JA, Glockner G, Rajandream MA, Sucgang R, Berriman M, Song J, Olsen R, Szafranski K, Xu Q (2005). The genome of the social amoeba *Dictyostelium discoideum*. Nature.

[6] King AJ, Cragg SM, Li Y, Dymond J, Guille MJ, Bowles DJ, Bruce NC, Graham IA, McQueen-Mason SJ (2010). Molecular insight into lignocellulose digestion by a marine isopod in the absence of gut microbes. Proc Natl Acad Sci U S A.

[7] Bayer EA, Belaich JP, Shoham Y, Lamed R (2004). The cellulosomes: multienzyme machines for degradation of plant cell wall polysaccharides. Annu Rev Microbiol.

[8•] Brunecky R, Alahuhta M, Xu Q, Donohoe BS, Crowley MF, Kataeva IA, Yang S-J, Resch MG, Adams MWW, Lunin VV (2013). Revealing Nature’s cellulase diversity: the digestion mechanism of *Caldicellulosiruptor bescii* CelA. Science.

[9•] Ekborg NA, Morrill W, Burgoyne AM, Li L, Distell DL (2007). CelAB a multifunctional cellulase encoded by *Teredinibacter turnerae* T7902(T), a culturable symbiont isolated from the wood-boring marine bivalve *Lyrodus pedicellatus*. Appl Environ Microbiol.

[10] Vaaje-Kolstad G, Westereng B, Horn SJ, Liu ZL, Zhai H, Sørlie M, Eijsink VGH (2010). An oxidative enzyme boosting the enzymatic conversion of recalcitrant polysaccharides. Science.

[11••] Beeson WT, Vu VV, Span EA, Phillips CM, Marletta MA (2015). Cellulose degradation by polysaccharide monooxygenases. Annu Rev Biochem.

[12] Agger JW, Isaksen T, Varnai A, Vidal-Melgosa S, Willats WGT, Ludwig R, Horn SJ, Eijsink VGH, Westereng B (2014). Discovery of LPMO activity on hemicelluloses shows the importance of oxidative processes in plant cell wall degradation. Proc Natl Acad Sci U S A.

[13•] Kjaergaard CH, Qayyum MF, Wong SD, Xu F, Hemsworth GR, Walton DJ, Young NA, Davies GJ, Walton PH, Johansen KS (2014). Spectroscopic and computational insight into the activation of O-2 by the mononuclear Cu center in polysaccharide monooxygenases. Proc Natl Acad Sci U S A.

[14] Arantes V, Goodell B, Nicholas DD, Goodell B, Schultz TP (2014). Current understanding of brown-rot fungal degradation mechanisms: a review. Biodeterioration and Protection of Sustainable Biomaterials.

[15] Bugg TDH, Ahmad M, Hardiman EM, Rahmanpour R (2011). Pathways for degradation of lignin in bacteria and fungi. Nat Prod Rep.

[16••] Pollegioni L, Tonin F, Rosini E (2015). Lignin-degrading enzymes. FEBS J.

[17] Floudas D, Binder M, Riley R, Barry K, Blanchette RA, Henrissat B, Martinez AT, Otillar R, Spatafora JW, Yadav JS (2012). The Paleozoic origin of enzymatic lignin decomposition reconstructed from 31 fungal genomes. Science.

[18••] Brune A (2014). Symbiotic digestion of lignocellulose in termite guts. Nat Rev Microbiol.

[19] Distel DL, Amin M, Burgoyne A, Linton E, Mamangkey G, Morrill W, Nove J, Wood N, Yang J (2011). Molecular phylogeny of Pholadoidea Lamarck, 1809 supports a single origin for xylotrophy (wood feeding) and xylotrophic bacterial endosymbiosis in Bivalvia. Mol Phylogenet Evol.

[20] Simmons CW, Reddy AP, D’haeseleer PD, Khdyakov J, Billis K, Pati A, Simmons BA, Singer SW, Thelen MP, van der Gheynst J (2014). Metatranscriptomic analysis of lignocellulolytic microbial communities involved in high-solids decomposition of rice straw. Biotechnol Biofuels.

[21] Scharf ME (2015). Omic research in termites: an overview and a roadmap. Front Genet.

[22] Boucias DG, Cai Y, Sun Y, Lietze V-U, Sen R, Raychoudhury R, Scharf ME (2013). The hindgut lumen prokaryotic microbiota of the termite *Reticulitermes flavipes* and its responses to dietary lignocellulose composition. Mol Ecol.

[23] Mikaelyan A, Strassert JFH, Tokuda G, Brune A (2014). The fibre-associated cellulolytic bacterial community in the hindgut of wood-feeding higher termites (*Nasutitermes* spp.). Environ Microbiol.

[24] Ahmad M, Roberts JN, Hardiman EM, Singh R, Eltis LD, Bugg TDH (2011). Identification of DypB from *Rhodococcus jostii* RHA1 as a lignin peroxidase. Biochemistry.

[25] Brown ME, Barros T, Chang MCY (2012). Identification and characterization of a multifunctional dye peroxidase from a lignin-reactive bacterium. ACS Chem Biol.

[26] Rahmanpour R, Bugg TDH (2015). Characterisation of Dyp-type peroxidases from *Pseudomonas fluorescens* Pf-5: oxidation of Mn(II) and polymeric lignin by Dyp1B. Arch Biochem Biophys.

[27] Majumdar S, Lukk T, Solbiati JO, Bauer S, Nair SK, Cronan JE, Gerlt JA (2014). Roles of small laccases from *Streptomyces* in lignin degradation. Biochemistry.

[28•] Gall DL, Kim H, Lu F, Donohue TJ, Noguera DR, Ralph J (2014). Stereochemical features of glutathione-dependent enzymes in the *Sphingobium* sp strain SYK-6 beta-aryl etherase pathway. J Biol Chem.

[29] Salvachua D, Karp EM, Nimlos CT, Vardon DR, Beckham GT (2015). Towards lignin consolidated bioprocessing: simultaneous lignin depolymerisation and product generation by bacteria. Green Chem.

[30] de Gannes V, Eudoxie G, Hickey WJ (2013). Prokaryotic successions and diversity in composts as revealed by 454-pyrosequencing. Bioresour Technol.

[31] Tian JH, Pourcher AM, Bouchez T, Gelhaye E, Peu P (2014). Occurrence of lignin degradation genotypes and phenotypes among prokaryotes. Appl Microbiol Biotechnol.

[32] Graham JE, Clark ME, Nadler DC, Huffer S, Chokhawala HA, Rowland SE, Blanch HW, Clark DS, Robb FT (2011). Identification and characterization of a multidomain hyperthermophilic cellulase from an archaeal enrichment. Nat Commun.

[33] Kataoka M, Ishikawa K (2014). A new crystal form of a hyperthermophilic endocellulase. Acta Crystallogr F Struct Biol Commun.

[34] Bjordal CG (2012). Evaluation of microbial degradation of shipwrecks in the Baltic Sea. Int Biodeterior Biodegr.

[35] Pedersen NB, Gierlinger N, Thygesen LG (2015). Bacterial and abiotic decay in waterlogged archaeological *Picea abies* (L.) Karst studied by confocal Raman imaging and ATR-FTIR spectroscopy. Holzforschung.

[36] Fagervold SK, Romano CF, Kalenitchenko D, Borowski C, Nunes-Jorge A, Martin D, Galand PE (2014). Microbial communities in sunken wood are structured by wood-boring bivalves and location in a submarine canyon. PLoS One.

[37] Kunii M, Yasuno M, Shindo Y, Kawata T (2014). A *Dictyostelium* cellobiohydrolase orthologue that affects developmental timing. Dev Genes Evol.

[38] Blifernez-Klassen O, Klassen V, Doebbe A, Kersting K, Grimm P, Wobbe L, Kruse O (2012). Cellulose degradation and assimilation by the unicellular phototrophic eukaryote Chlamydomonas reinhardtii. Nat Commun.

[39] Toulza E, Shin M-S, Blanc G, Audic S, Laabir M, Collos Y, Claverie J-M, Grzebyk D (2010). Gene expression in proliferating cells of the dinoflagellate *Alexandrium catenella* (Dinophyceae). Appl Environ Microbiol.

[40] Blackman LM, Cullerne DP, Hardham AR (2014). Bioinformatic characterisation of genes encoding cell wall degrading enzymes in the *Phytophthora parasitica* genome. BMC Genomics.

[41••] Riley R, Salamov AA, Brown DW, Nagy LG, Floudas D, Held BW, Levasseur A, Lombard V, Morin E, Otillar R (2014). Extensive sampling of basidiomycete genomes demonstrates inadequacy of the white-rot/brown-rot paradigm for wood decay fungi. Proc Natl Acad Sci USA.

[42••] Busk PK, Lange L (2015). Classification of fungal and bacterial lytic polysaccharide monooxygenases. BMC Genomics.

[43] Eastwood DC, Floudas D, Binder M, Majcherczyk A, Schneider P, Aerts A, Asiegbu FO, Baker SE, Barry K, Bendiksby M (2011). The plant cell wall-decomposing machinery underlies the functional diversity of forest fungi. Science.

[44] Shelomi M, Watanabe H, Arakawa G (2014). Endogenous cellulase enzymes in the stick insect (*Phasmatodea*) gut. J Insect Physiol.

[45] Tsuji A, Tominaga K, Nishiyama N, Yuasa K (2013). Comprehensive enzymatic analysis of the cellulolytic system in digestive fluid of the sea hare *Aplysia kurodai*. Efficient glucose release from sea lettuce by synergistic action of 45 kDa endoglucanase and 210 kDa beta-glucosidase. PLoS One.

[46] Bui THH, Lee SY (2015). Endogenous cellulase production in the leaf litter foraging mangrove crab *Parasesarma erythodactyla*. Comp Biochem Physiol B Biochem Mol Biol.

[47] Kostanjsek R, Milatovic M, Srus J (2010). Endogenous origin of endobeta-1,4-glucanase in common woodlouse *Porcellio scaber* (Crustacea Isopoda). J Comp Physiol B Biochem Syst Environ Physiol.

[48] Nozaki M, Miura C, Tozawa Y, Miura T (2009). The contribution of endogenous cellulase to the cellulose digestion in the gut of earthworm (*Pheretima hilgendorfi*: Megascolecidae). Soil Biol Biochem.

[49] Betcher MA, Fung JM, Han AW, O’Connor R, Seronay R, Concepcion GP, Distel DL, Haygood MG (2012). Microbial distribution and abundance in the digestive system of five shipworm species (Bivalvia: Teredinidae). PLoS One.

[50••] O’Connor RM, Fung JM, Sharp KH, Benner JS, McClung C, Cushing S, Lamkin ER, Fomenkov AI, Henrissat B, Londer YY (2014). Gill bacteria enable a novel digestive strategy in a wood-feeding mollusk. Proc Natl Acad Sci USA.

[51] Lechene CP, Luyten Y, McMahon G, Distel DL (2007). Quantitative imaging of nitrogen fixation by individual bacteria within animal cells. Science.

[52] Sakamoto K, Toyohara H (2009). Putative endogenous xylanase from brackish-water clam *Corbicula japonica*. Comp Biochem Physiol B Biochem Mol Biol.

[53] Koenig H, Li L, Froehlich J (2013). The cellulolytic system of the termite gut. Appl Microbiol Biotechnol.

[54] Jaenicke E, Fraune S, May S, Irmak P, Augustin R, Meesters C, Decker H, Zimmer M (2009). Is activated hemocyanin instead of phenoloxidase involved in immune response in woodlice?. Dev Comp Immunol.

[55] Allardyce BJ, Linton SM (2008). Purification and characterisation of endo-beta-1,4-glucanase and laminarinase enzymes from the gecarcinid land crab *Gecarcoidea anatalis* and the aquatic crayfish *Cherax destructor*. J Exp Biol.

[56] Tsukagoshi H, Nakamura A, Ishida T, Touhara KK, Otagiri M, Moriya S, Samejima M, Igarashi K, Fushinobu S, Kitamoto K (2014). Structural and biochemical analyses of glycoside hydrolase family 26 beta-mannanase from a symbiotic protist of the termite *Reticulitermes speratus*. J Biol Chem.

[57] Zheng H, Dietrich C, Thompson CL, Meuser K, Brune A (2015). Population structure of endomicrobia in single host cells of termite gut flagellates (*Trichonympha* spp.). Microb Environ.

[58] Poulsen M, Hu H, Li C, Chen Z, Xu L, Otani S, Nygaard S, Nobre T, Klaubauf S, Schindler PM (2014). Complementary symbiont contributions to plant decomposition in a fungus-farming termite. Proc Natl Acad Sci U S A.

[59•] Kern M, McGeehan JE, Streeter SD, Martin RNA, Besser K, Elias L, Eborall W, Malyon GP, Payne CM, Himmel ME (2013). Structural characterization of a unique marine animal family 7 cellobiohydrolase suggests a mechanism of cellulase salt tolerance. Proc Natl Acad Sci U S A.

[60] Vispo C, Hume ID (1995). The digestive tract and digestive function in the North American porcupine and beaver. Can J Zool.

[61] Zhu L, Wu Q, Dai J, Zhang S, Wei F (2011). Evidence of cellulose metabolism by the giant panda gut microbiome. Proc Natl Acad Sci U S A.

[62] Xue Z, Zhang W, Wang L, Hou R, Zhang M, Fei L, Zhang X, Huang H, Bridgewater LC, Jiang Y (2015). The bamboo-eating giant panda harbors a carnivore-like gut microbiota, with excessive seasonal variations. mBio.

[63] McDonald R, Schreier HJ, Watts JEM (2012). Phylogenetic analysis of microbial communities in different regions of the gastrointestinal tract in *Panaque nigrolineatus*, a wood-eating fish. PLoS One.

[64] McDonald BR, Zhang F, Schreier HJ, Watts JEM (2015). 1: Nitrogenase diversity and activity in the gastrointestinal tract of the wood-eating catfish *Panaque nigrolineatus*. ISME J.

[65•] Blumer-Schuette SE, Alahuhta M, Conway JM, Lee LL, Zurawski JV, Giannone RJ, Hettich RL, Lunin VV, Himmel ME, Kelly RM (2015). Discrete and structurally unique proteins (Tapirins) mediate attachment of extremely thermophilic caldicellulosiruptor species to cellulose. J Biol Chem.

[66] Resch MG, Donohoe BS, Baker JO, Decker SR, Bayer EA, Beckham GT, Himmel ME (2013). Fungal cellulases and complexed cellulosomal enzymes exhibit synergistic mechanisms in cellulose deconstruction. Energy Environ Sci.

[67] Eibinger M, Ganner T, Bubner P, Rosker S, Kracher D, Haltrich D, Ludwig R, Plank H, Nidetzky B (2014). Cellulose surface degradation by a lytic polysaccharide monooxygenase and its effect on cellulase hydrolytic efficiency. J Biol Chem.

[68•] Li X, Jackson P, Rubtsov DV, Faria-Blanc N, Mortimer JC, Turner SR, Krogh KB, Johansen KS, Dupree P (2013). Development and application of a high throughput carbohydrate profiling technique for analyzing plant cell wall polysaccharides and carbohydrate active enzymes. Biotechnol Biofuels.

[69] Larkin MA, Blackshields G, Brown NP, Chenna R, McGettigan PA, McWilliam H, Valentin F, Wallace IM, Wilm A, Lopez R, Thompson JD, Gibson TJ, Higgins DG (2007). ClustalW and ClustalX version 2. Bioinformatics.

[70] Robert X, Gouet P (2014). Deciphering key features in protein structures with the new ENDscript server. Nucleic Acids Res.

[71] Biasini M, Bienert S, Waterhouse A, Arnold K, Studer G, Schmidt T, Kiefer F, Gallo TG, Bertoni M, Bordoli L, Schwede T (2014). SWISS-MODEL: modelling protein tertiary and quaternary structure using evolutionary information. Nucleic Acids Res.

[72] Knott BC, Haddad Momeni M, Crowley MF, Mackenzie LF, Gőtz AW, Sandgren M, Withers SG, Ståhlberg J, Beckham GT (2014). The mechanism of cellulose hydrolysis by a two-step, retaining cellobiohydrolase elucidated by structural and transition path sampling studies. J Am Chem Soc.

[73] Li L, Li C, Sarkar S, Zhang J, Witham S, Zhang Z, Wang L, Smith N, Petukh M, Alexov E (2012). DelPhi: a comprehensive suite for DelPhi software and associated resources. BMC Biophys.

[74] Arantes V, Jellison J, Goodell B (2012). Peculiarities of brown-rot fungi and biochemical Fenton reaction with regard to their potential as a model for bioprocessing biomass. Appl Microbiol Biotechnol.

